# Genotyping of Chlamydia abortus using multiple loci variable number of tandem repeats analysis technique

**DOI:** 10.1186/s12917-022-03142-6

**Published:** 2022-01-24

**Authors:** Sara Barati, Naghmeh Moori Bakhtiari, Leili Shokoohizadeh, Masoud Ghorbanpoor, Hassan Momtaz

**Affiliations:** 1 Post graduate of Veterinary Medicine school, Shahid Chamran University of Ahvaz, Ahvaz, Iran; 2 Department of Pathobiology, Faculty of Veterinary Medicine, Shahid Chamran University of Ahvaz, Ahvaz, Iran; 3 Department of Microbiology, Faculty of Medicine, Hamadan university of Medical Sciences, Hamadan, Iran; 4 Department of Pathobiology, Faculty of Veterinary Medicine, Islamic Azad University of Shahrekord, Shahrekord, Iran

**Keywords:** *Chlamydia abortus*, Genotyping, Iran, MLVA, Ruminants

## Abstract

**Background:**

The correlation between various factors (geographical region, clinical incidence, and host type) and the genomic heterogeneity has been shown in several bacterial strains including *Chlamydia abortus.*

**Methods:**

The aim of this study was to survey the predominant types of *C. abortus* strains isolated from ruminants in Iran by the multiple loci variable number of tandem repeats (VNTR) analysis (MLVA) method. *C. abortus* infection was evaluated in a total of 117 aborted fetuses by real-time PCR. The isolation was done via the inoculation of the positive samples in chicken embryo and the L929 cell line. Genotyping was carried out by MLVA typing technique.

**Results:**

Forty samples (34.2%) were detected with *C. abortus* infection; however, chlamydial infection in ruminants of Charmahal/Bakhtiari (3 bovines and 35 sheep) was higher than that of Khuzestan (2 sheep). All MLVA types (MT1-MT8) were detected in the collected samples from Charmahal/Bakhtiari but only 2 types (MT1 and MT3) were reported in samples from Khuzestan. The main MT type was MT1 (32% of aborted fetuses). Although in this study only 9 cow samples were investigated, they possessed similar clusters to those obtained from sheep (MT1 and MT6).

Variation of type in sheep samples (MT1 to MT8) was more than that of bovine samples (MT1, and MT6).

**Conclusion:**

By this research revealed that *C.abortus* was responsible for a significant percentage of ruminant abortion in two studied regions. The main MT type was MT1 (32% of aborted fetuses) and also 7 different genotypes were involved in infections. So it is concluded that diversity in *C.abortus* genotyping is high in two regions.

## Background

The family of *Chlamydiaceae* contains obligate intracellular Gram-negative bacteria, with 14 confirmed species (*Chlamydia trachomatis, Chlamydia pneumoniae, Chlamydia abortus, Chlamydia caviae, Chlamydia felis, Chlamydia muridarum, Chlamydia pecorum, Chlamydia psittaci, Chlamydia suis, Chlamydia avium, Chlamydia gallinacea, Chlamydia serpentis, Chlamydia buteonis and Chlamydia poikilothermis)* and 4 candidate species (*Chlamydia ibidis, Chlamydia corallus, Chlamydia sanzinia* and *Chlamydia testudinis)* related to a single genus of *Chlamydia* [1–3].

*C. abortus* is the most common cause of infectious abortion in sheep and goats in many countries in the world. Also, it recognized as causative agent of a various diseases, including fertility disorder, conjunctivitis, arthritis, mastitis, and pulmonary inflammation (endemic among small ruminants) [4–7]. No clinical signs are observed in the pregnant animals prior to the abortion (in last 2–3 weeks of gestation) or the delivery of very weak lambs. Moreover, the disease affects goats and to a lesser degree, cattle, horses, pigs, and deer; however, little is known about the rate of chlamydial infections because of the lack of epidemiological evidence [8]. Diagnosis of chlamydial infections can be attempted by various detection methods, including culture, antigen detection, serology, and nucleic acid amplification. However, in spite of various available methods (e.g. staining and microscopic examination, isolation, molecular and serological diagnosis) the detection of Chlamydiae in clinical specimens remains a major challenge and need to high experiences [9]. This bacterium is a highly homogeneous species with low genetic heterogeneity. *C. abortus* as the recently emerged species, is closely related to more diverse *C. psittaci* with more diversity in genetics, host association and disease pathology. Also, gene-flow could be occurred between *C. psittaci* and *C. abortus* during host cell coinfection with these two species [10, 11]. Gene exchanging by bacteriophage is probable since some bacteriophages have been identified for *C. abortus* and *C. psittaci* [12]. Gene diversity within the genus is much lower in *C. abortus* compared to other species; moreover, strong geographical signatures within phylogeny were reported [13]. By sequencing of *C. abortus* core genome, several major regions with high variability observed which encode highly variable proteins such as TMH (transmembrane head protein) and Pmp (polymorphic membrane protein) families. These variable loci are source of diversity in disease causation and host tropism in these bacteria [14, 15].

Multiple loci variable number of tandem repeats analysis (MLVA) is a method which utilizes the naturally occurring variation in the number of tandems repeated in a set of variable number of tandem repeats (VNTR) loci found in the genome of most bacterial species [16]. The genomic study of bacteria is an applicable approach not only to detection of infection sources and resistance to antibiotics, but also to studying the relationship of genomic profile with the intensity and type of clinical signs and host tropism [17–19]. This method has been used successfully for *C. abortus* and *C. psittaci* typing, the causative agents of ovine and avian chlamydiosis, respectively. The recently developed MLVA system is capable of classifying a panel of 111 *C. abortus* isolates into 6 genotypes based on five VNTR loci and their respective PCR fragments [6]. Also, 38 reference strains of *C. psittaci* with 5 selected VNTR primers as well as 6 *C. abortus* prototype strains with the 8 VNTR primers were established for type *C. psittaci* [20]. The isolation of each pathogen could be useful in experimental studies for the identification of pathogenesis or production of vaccines. Until now, many serological and molecular studies have been conducted on *C. abortus* infections; despite this, they have been unsuccessful in isolating it. Also, no study has reported the genetic heterogeneity in *C. abortus* isolates in Iran. Therefore, the present study aimed at the isolation and identification of genetic heterogeneity among *C. abortus* strains in the ruminants of Iran. Results of this research could act as a basis for epidemiological and control programs in Iran.

## Methods

A schematic of the protocol we developed to study of *C. abortus* MLVA typing in aborted fetus is shown in below.

liver, spleen, and abomasum of aborted fetus in SPG transport medium.

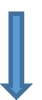



DNA extraction and *C.abortus* infection detection by real time PCR and conventional PCR.

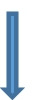



MLVA typing of all positive samples **/** Isolation by Yolk sac inoculation of some of positive samples.

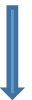

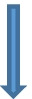





Confirmation of *C.abortus* infection in chicken emberyo by conventional PCR and staining.

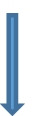



Inoculation of infected yolk sac to L929 cell line.



Confirmation of *C.abortus* infection in cell line by staining, conventional PCR, fluorescent Ab, immunodot.

### Sample collection

**Fig. 1:**
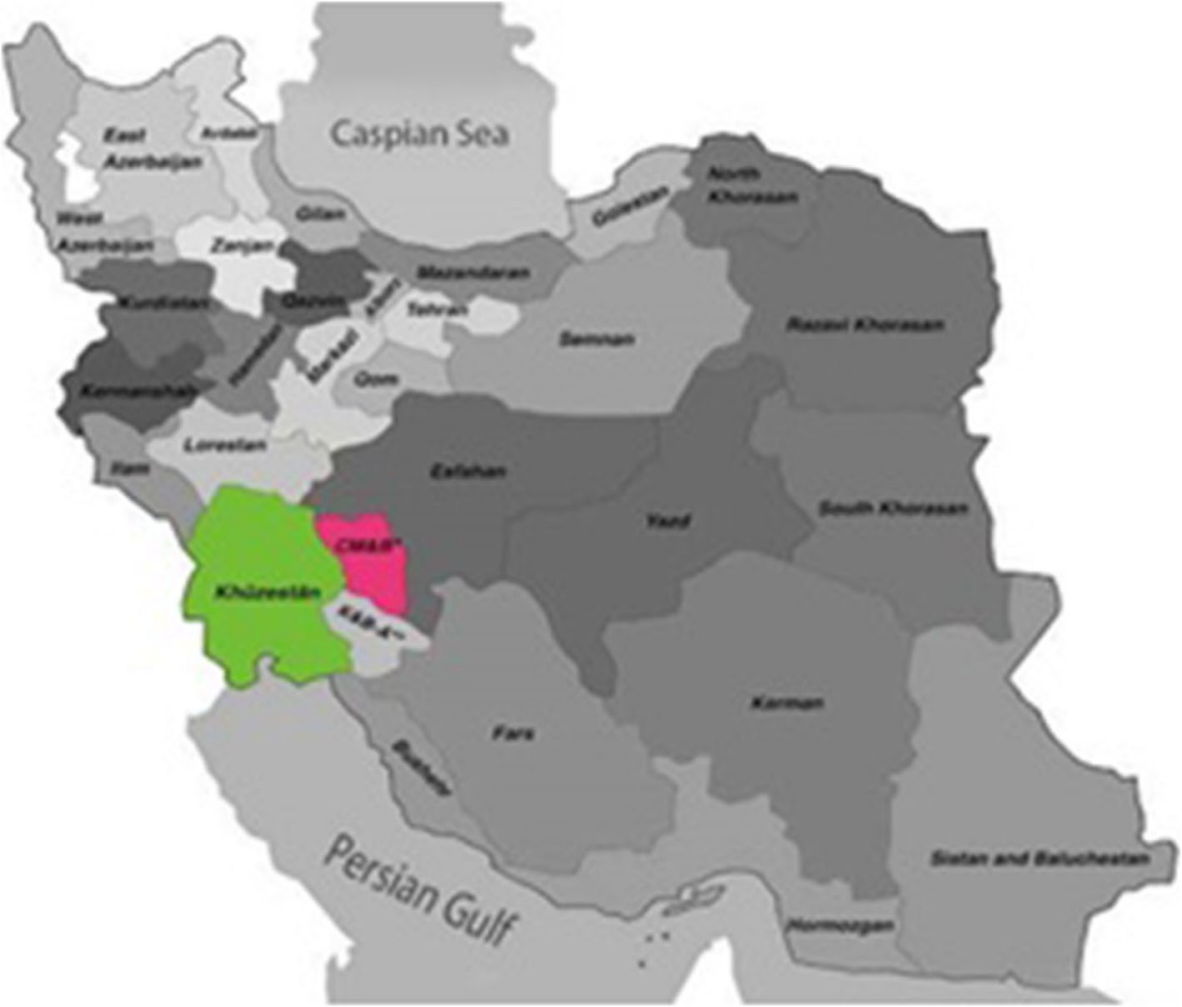
Demonstration of geographical situation of two studied provinces

A total of 117 aborted fetuses (9 cattle and 108 sheep from Khuzestan and Chaharmahal/ Bakhtiari provinces) were collected from different herds located in the south-west of Iran (from 2014 to 2016). Samples (liver, spleen, and abomasum) were collected from the aborted fetuses and kept in Sucrose Phosphate Glutamate (SPG) transport medium and settled at − 70 °C. Strict aseptic protocols were used for each sample to avoid cross-contamination. Two sampling provinces are demonstrated in Fig. 1.

### Detection of *C. abortus* infected samples

Chlamydial infection of all studied samples was evaluated by *Chlamydiales*-specific PCR [21]. Genomic DNA was extracted from each sample using commercial DNA extraction Kit (CinaGen, Iran) in accordance to the manufacturer’s instructions.

The PCR assay was performed in a total volume of 25 μL, includ ing bacterial DNA (5 μL), forward and reverse primers (1 μL; 0.1 μM), 2X PCR Master Mix (12.5 μL; Ampliqon), and nuclease-free water (5.5 μL). The temperature of the protocol was 94 °C for 10 min (primary denaturation), 35 cycles of 94 °C for 30 s and 48 °C for 30 s. Nuclease-free water as negative control and *C. abortus* s26/3 as positive control. Primers sequence and aim gene (16 s–23 s spacer region) were shown in Table 1.

**Table 1:** The sequence of primers of *Chlamydiales* and *C. abortus* used in the PCR assay

References	Length (bp)	Sequence (5′–3′)	Genes
Borel et al, 2006 [21]	352 bp	Forward 5′-CAA GGT GAG GCT GAT GAC-3′ Reverse 5′-TCG CCT KTC AAT GCC AAG-3’	*Chlamydiales* 16 s–23 s spacer region (**100pmoles/ ul**)
Longbottom et al 2002 [22]	222 bp	Forward 5’- TTTTCAGGATCCTATTGTCCTCCAGGCA −3′ Reverse 5′- GTGTCGACATCAGCATAAATAGCCCCG−3′	*Chlamydia. abortus* *pmp1* gene (**100 pmoles/ ul**)

*C. abortus* infection in the positive samples from the primary stage was detected by real-time PCR designed by Panchev et al. (2009) based on the presence of *omp*A gene. The *C. abortus* s26/3 and *C. psittaci* 6 BC used as the positive controls, with nuclease free water serving as the negative control.

The Real-time PCR reaction was carried out in final volume of 25 μL by the Biosystem step one (ABI) device. Each reaction was duplex and consist of 3 μL of extracted DNA, 1 μL of each primer (final concentration of 0.6 μM), 2 μL of TaqMan probe (0.3 μM concentration). The temperature of the protocol was 95 °C for 10 min (primary denaturation), 45 cycles of 95 °C for 15 s and 60 °C for 45 s [23]. Primers sequence and probs were shown in Table 2.

**Table 2:** Primers and TaqMan probes used for detection of *C. abortus* and internal amplification control by real-time PCR

Name	Sequence	Amplicon size (bp)
CpaOMP1-F CpaOMP1-R CpaOMP1-Sa	5-GCAACTGACACTAAGTCGGCTACA-3 5-ACAAGCATGTTCAATCGATAAGAGA-3 FAMb- TAAATACCACGAATGGCAAGT TGGTTTAGCG-TAMRAc	82
EGFP-1-F EGFP-10-R EGFP-HEXd	5-GACCACTACCAGCAGAACAC-3 5-CTTGTACAGCTCGTCCATGC-3 HEXd- AGCACCCAGTCCGCCCTGAGCABHQ1 e	177

^a^ Conventional TaqMan probe

^b^ FAM 6-carboxy-fluorescein

^c^ TAMRA 6-carboxy-tetramethylrhodamin

^d^ HEX hexachlorofluorescein

^e^ Black hole quencher

### Multi-locus VNTR analysis (MLVA)

MLVA genotyping was carried out by targeting five tandem repeat loci (ChlaAb_457, ChlaAb_581, ChlaAb_620, Chla Ab_914 and ChlaAb_300) as described previously [6]. Initially, PCR was performed on the *C. abortus* S26/3 strain as the control.

For the VNTR amplifications, PCR was performed in a total volume of 25 mL with 1 U of Hot Start Taq DNA polymerase (CinnaGen, Iran). The sequence of the designed VNTR primers based on the whole genomic sequence of *C. abortus* S26/3 is shown in Table 3. Five microliters of the amplification product were loaded in 4% agarose gel. The agarose gel was colored with safe-stain (3–5 μL/100 cc) (CinnaGen, Iran) and was then visualized under UV light and photographed.

**Table 3:** Primer sequences for detection of five tandem repeat loci for genotyping of *C. abortus* using MLVA method

Name	Forward primer (5–30)	Reverse primer (50–30)	Repeat unit (bp)	repetition number	size
ChlaAb_300	AGACCTAAAGCGCCACCTTCA	ATGCGCCAATCTATACGCTGA	9	3.0	
ChlaAb_457	GTACAAAAAAAACGTAGCAGCAAGAA	CACGTTGGCAAGAAGCTGTGT	28	2.8	358
ChlaAb_581	ACAGCACCAGCATTAGCCG	TGGATAGTTGTCGCTGGTGG	15	2.1	161
ChlaAb_620	ATGCTATAATTGCTTAGTTTTTTTAACATTG	CACATGCCGCCCTGAAC	11	3.6	163
ChlaAb_914	TTTAAAGTTTCCGTATCTTTGTAATCGAT	TTTTAGAATTCGCATCATTACCAGAA	15	2.3	174

### Isolation and identification

A number of positive samples (5 samples of 5 aborted fetus) from the previous stage were selected for inoculation in chicken embryos (eggs were incubated at 37.8 °C and 5% CO_2_ with a relative humidity of 65% for 7 days). Before yolk sac inoculation, samples (liver, spleen, and abomasum secretion) were homogenized (1% in sterile PBS) and treated with different antibiotics, such as vancomycin (1 mg/mL), gentamycin (200 μg/mL), and amphotericin B (50 μg/mL) and incubated at 4 °C for 24 h [24]. After inoculating 0.2 mL of the treated sample, the eggs were incubated in the conditions mentioned above for a 12-day post-infection (12 dpi). Eggs with embryonic mortality after 4 days of inoculation were evaluated to detect chlamydial inclusion bodies in the yolk sac cells by different staining methods such as Giemsa and Ziehl Neelsen and PCR assay [22, 25]. The confirmed positive samples by two staining methods and *C. abortus* specific PCR, were cultured in an L929 cell line (Pasteur Institute-Iran). For the development of a monolayer cell with the proper density (1 × 10^5^ cell/mL), the RPMI 1640 medium (Bioidea- Iran) supplemented with 10% fetal bovine serum (Bioidea, Iran), vancomycin (100 mg/mL), gentamycin (50 μg/mL) and amphotericin B (50 μg/mL) (Sigma, USA) was utilized. The cell line was inoculated with 500 μL of the yolk sac specimen from the primary stage and incubated for 3 days at 37.8 °C and 5% CO_2_ with a relative humidity of 65%. A monolayer cell culture without inoculation was used as the negative control culture. After 48 h of inoculation, the monolayer was trypsinized and a smear was prepared from the collected cells and was then stained with Giemsa [25] and Modified Macchiavello [26] to reveal the inclusion bodies and be confirmed by PCR assay [22]. Confirmation of Chlamydiae infection in chicken embryo and L929 cell line was conducted by PCR. Thereby, each reaction was carried out in a final volume of 25 μL containing 1× PCR buffer, 0.5 μM of each primer set, 200 μM of the four deoxynucleoside triphosphates (dNTPs), 2 mM MgCl_2_, and 0.5 U of Taq polymerase (SinaGen, Iran). The primers used for the detection of *Chlamydiales and C. abortus* are shown in Table 1. By means of these primers, 16 s–23 s spacer regions in *chlamydia* spp. and *pomp*90–3 gene in *C. abortus* could be detected. PCR reactions were performed in an Eppendorf thermo-cycler (Eppendorf, Germany) under the following conditions: initial denaturation for 10 min at 94 °C; 35 cycles of 30 s at 94 °C, annealing for 30 s at 48 °C (for 16 s–23 s spacer region and *pomp*90–3 genes), extension for 45 s at 72 °C and final extension step at 72 °C for 10 min. The PCR products were subjected to electrophoresis for 1 h at 70 V in 1.5% agarose gel with 3-5 μL/100 cc of safe-stain (CinnaGen, Iran). The results were visualized and photographed under ultraviolet illumination.

### Indirect fluorescent antibody test

The *C. abortus* infection of cells was confirmed by the indirect fluorescent antibody assay. For the preparation of an in-house anti-chlamydial antibody, 2 healthy female Albino rabbits were purchased from the experimental animal house of the related university and infected several times with the supernatant of *C. abortus* cell culture. Rabbits were retained under standard laboratory conditions and had free access to food pellets (Pars Animal Feed Co, Tehran, Iran) and tap water. Experiments were conducted according to the guide for the care and use of laboratory animals by the National Academy of Sciences of shahid chamran university, license no: EE/1400.3.02.32646/scu.ac.ir.

The monolayers of L929 cells on coverslips were infected with 100 μL of positive samples and incubated for 48 h at 38 °C with 5% CO2. Subsequently, fixation was carried out by cold methanol for 20 min at room temperature and coverslips were dried and blocked with 1% BSA (Bovine serum albumin) diluted in PBS. After 1 h of incubation at 37 °C in a humid chamber, coverslips were washed with PBST. Prior to being subjected to polyclonal rabbit anti-chlamydia-specific serum, coverslips were incubated with 0.1% Triton-100 for 20 min at room temperature. Anti-chlamydia serum was diluted 1/100 in PBS+ 1% BSA and incubated together with the infected cells on the coverslips for 1 h at 37 °C. Then, they were washed with PBST 4 times. Fluorescein isothiocyanate-labeled anti-rabbit antibody (Sigma-Aldrich, F9887) was diluted 1/200 in PBST (PBS + 0.2% Tween20) plus 1% BSA and incubated together with coverslips for 1 h at 37 °C. After 3 times of washing, the inclusion of the infected cells was observed using an IX-71 M fluorescence microscope (Olympus, Japan) at 4 and 40 × 0.3-fold magnification. Uninfected cells were used as the negative control in this method.

### Immunodotting assay

In the current experiment, 0.1 μL of different samples (V; *C. felis* vaccine, S; immune rabbit serum as positive control, A1 and A2; supernatant concentration of infected cell culture with *C. abortus* after 48 and 72 h, respectively, F; supernatant concentration of infected cell culture with *C. felis*, N; supernatant concentration of uninfected cell culture as negative control) were placed on nitrocellulose membrane. After drying at room temperature, the membrane was blocked with PBS + 0.2% Tween 20 for 2 h. Polyclonal rabbit anti-chlamydia-specific serum was diluted (1/200) in PBST (PBS+ 0.05% Tween 20) and incubated for 1 h. After washing 3 times with PBST, the mixture was adjusted with diluted (1/1000) horseradish peroxidase anti-rabbit antibody (Sigma-Aldrich, A0545) in PBST for 1 h. It was then washed with PBST 3 times and placed in chromogen substrate (H_2_O_2_ and 4-Chloro-1- Naphthol) for 15 min at room temperature. Room temperature and shaking were used in all stages.

### Data analysis

Data analysis was performed after carrying out the PCR of the VNTR loci and subsequent to determining the size of the PCR products on agarose gel via either capillary systems or an automated DNA sequence. The assessed PCR product size was used to calculate the number of repeated units in each locus. The flanking regions with known sizes were subtracted from the PCR product size which resulted in the net size of the repeat region. This size was divided by the repeated unit size which eventually revealed the number of repeats. There was a negligible inaccuracy in sizing, but for most of the parts, it did not prevent the assessment of the number of repeats [27]. Amplicons size was determined manually using a 100 bp ladder (CinnaGen, Iran). Based on the differences in the number and weight of the fragments, genotypes were assigned following MLVA fingerprint analysis. The bands or fragments were compared using the Dice method and clustered by the Unweighted Pair-Group Method with Arithmetic Mean (UPGMA) by referring to insilico.ehu.es online data analysis service.

## Results

Generally, Chlamydial infection was detected in 40 samples (34.1%) by order-specific conventional PCR. The infected samples in the 2 studied provinces consisted of 37 sheep and 3 cows. The number of positive samples in Charmahal/Bakhtiari (38/61) was more than that in Khuzestan (2/56) province. By species- specific real-time PCR, *C. abortus* infection was confirmed in all of them and coinfection was reported in any of the 40 studied samples. Results of primary selection of infected samples by *Chlamydiales*-specific PCR are shown in Table 4.

**Table 4:** Results of primary selection of infected samples by *Chlamydiales*-specific PCR

Host type	Positive		Negative		Prevalence CI 95%
	sheep	cow	sheep	cow	
Khuzestan	2 (3.7%)	0 (0%)	54 (96.42%)	0 (0%)	3.57% (0.048–0.013)
Charmahal/bakhtiari	35 (67.3%)	3 (33.33%)	17 (32.69)	6 (66.66%)	62.29% (0.742–0.498)
total	37	3	71	6	34% (0.426–0.254)

**Fig. 2:**
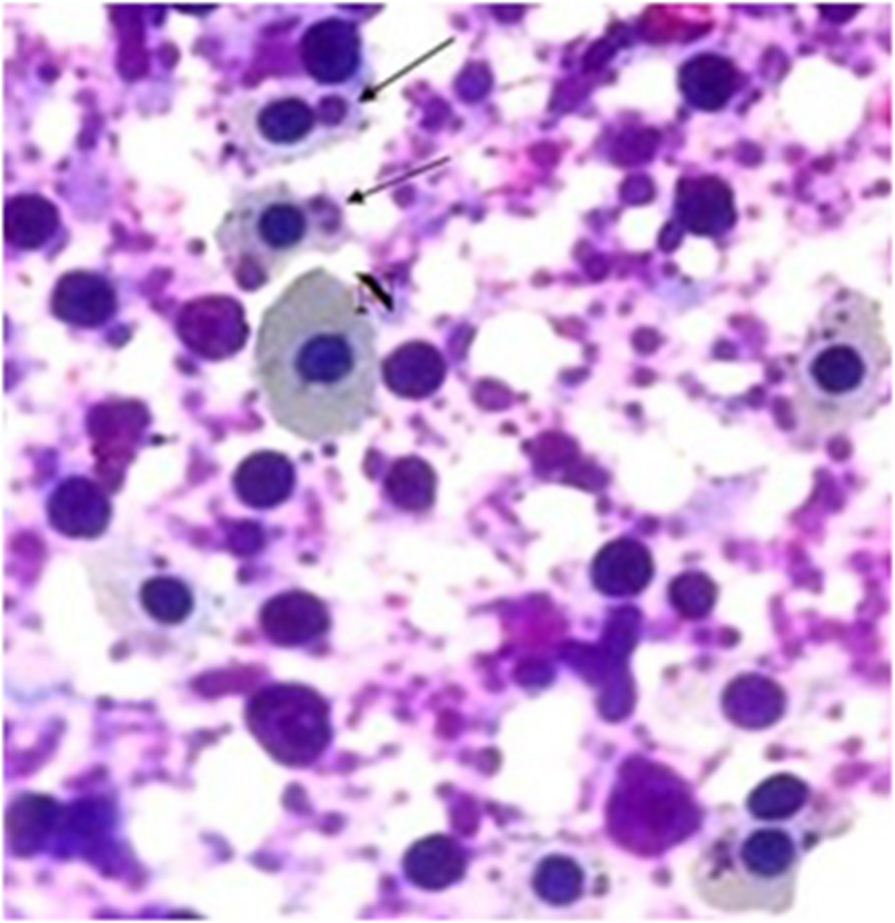
Giemsa staining of inoculated egg yolk sac with positive sample after 72 h (ED11). N and arrows demonstrate normal cells and inclusion in the infected cells, respectively

**Fig. 3:**
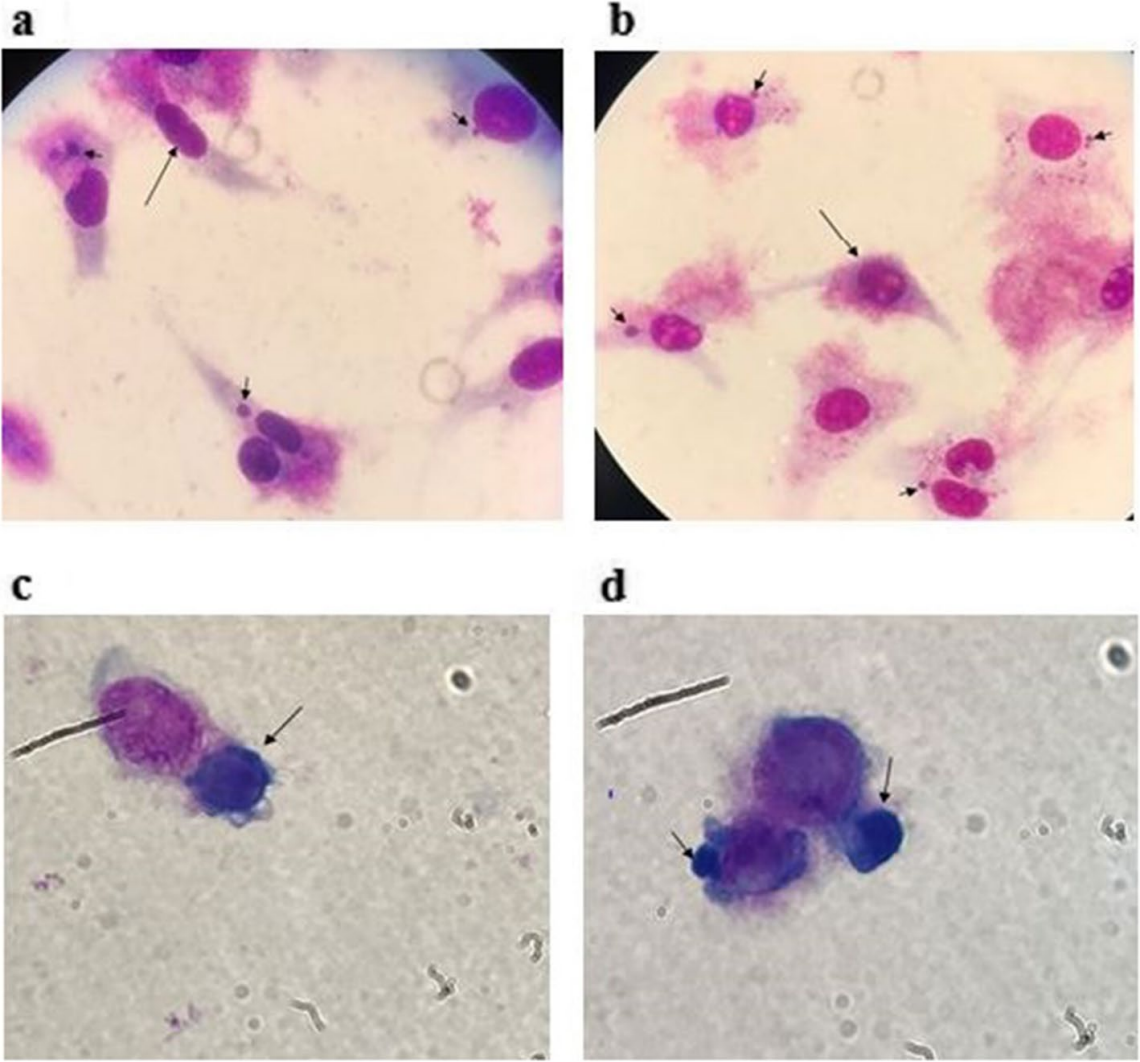
Morphology of *C. abortus* inclusion bodies (arrows) in L929 cells × 100. a & b; Giemsa staining of non- trypsinated infected cells; the long and short arrows show the normal and infected cells with inclusion, respectively. C & d; Giemsa staining of inclusion bodies (arrows) in trypsinated *C. abortus* infected cells

**Fig. 4:**
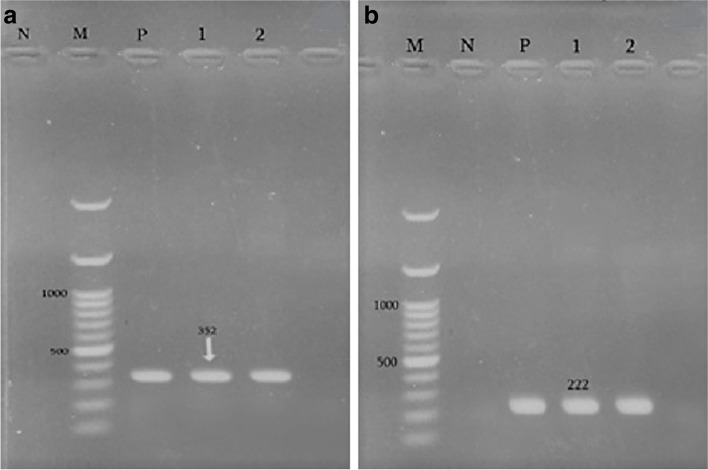
(**A**) PCR results with *Chlamydiales* specific primers cIGS-1f/ cIGS-1r in agarose gel N– negative control; M - molecular mass standard 100 bp ladder; P– positive control (352 bp); lines 1 and 2- tested samples. (**B**) PCR results with *C. abortus pomp*90–3 specific primers. N– negative control; P – positive control (222 bp); Lines 1–3 tested samples; M - molecular mass standard 100 bp ladder.

For isolation, after 1 passage of each positive sample in the chicken embryo, we demonstrated cytoplasmic inclusions in the yolk sac membrane of dead fetus (Fig. 2). By culturing the primary infected yolk sac in an L929 cell line, various sizes of dense cytoplasmic inclusions were detected in trypsinized and non trypsinized infected cell stained smears (Fig. 3). Also, *C. abortus* infection in the cell line was confirmed by producing 222 bp fragments by species-specific conventional PCR (Fig. 4).

**Fig. 5:**
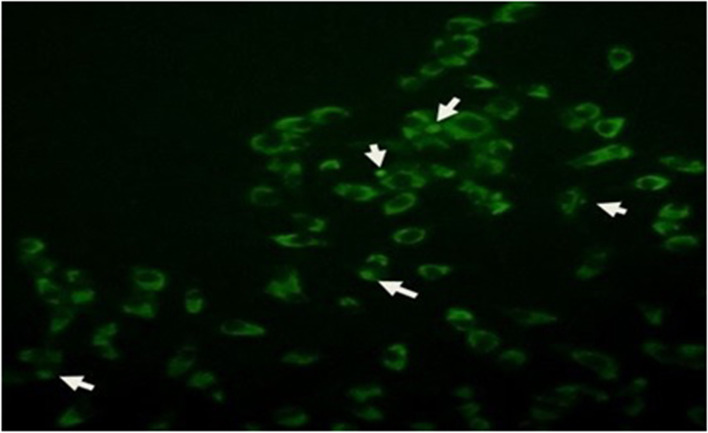
Indirect fluorescent antibody staining of non trypsinated *C. abortus* infected cells; Inclusion and infected cells are demonstrated with arrow

Cell infection was confirmed by indirect fluorescent antibody test and immunodotting assay. The manifestation of different sizes and densities of fluorescent cytoplasmic inclusions was a sign of cell infection as shown in Fig. 5. As regards the immunodotting assay, a strong reaction of V (*C. felis* vaccine), S (immune rabbit serum as positive control), A1(supernatant concentration of infected cell culture with *C. abortus* after 48 h), A2 (supernatant concentration of infected cell culture with *C. abortus* after 72 h) and F (supernatant concentration of infected cell culture with *C. felis*) samples with polyclonal rabbit anti-chlamydia-specific serum was observed although no reaction with the N (supernatant concentration of uninfected cell culture as negative control) sample was detected.

**Fig. 6:**
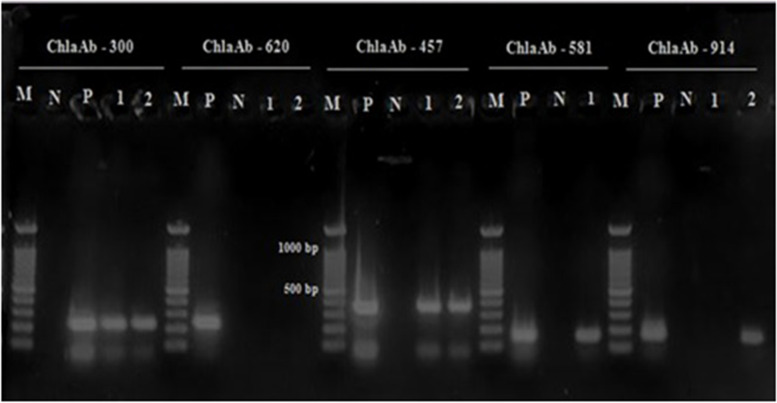
MLVA profile of *C. abortus* in 2 studied samples. M - Molecular mass standard 100 bp ladder; N-negative control; P- *C. abortus* S26/3 as positive control; lines 1 and 2- tested samples .

**Fig. 7:**
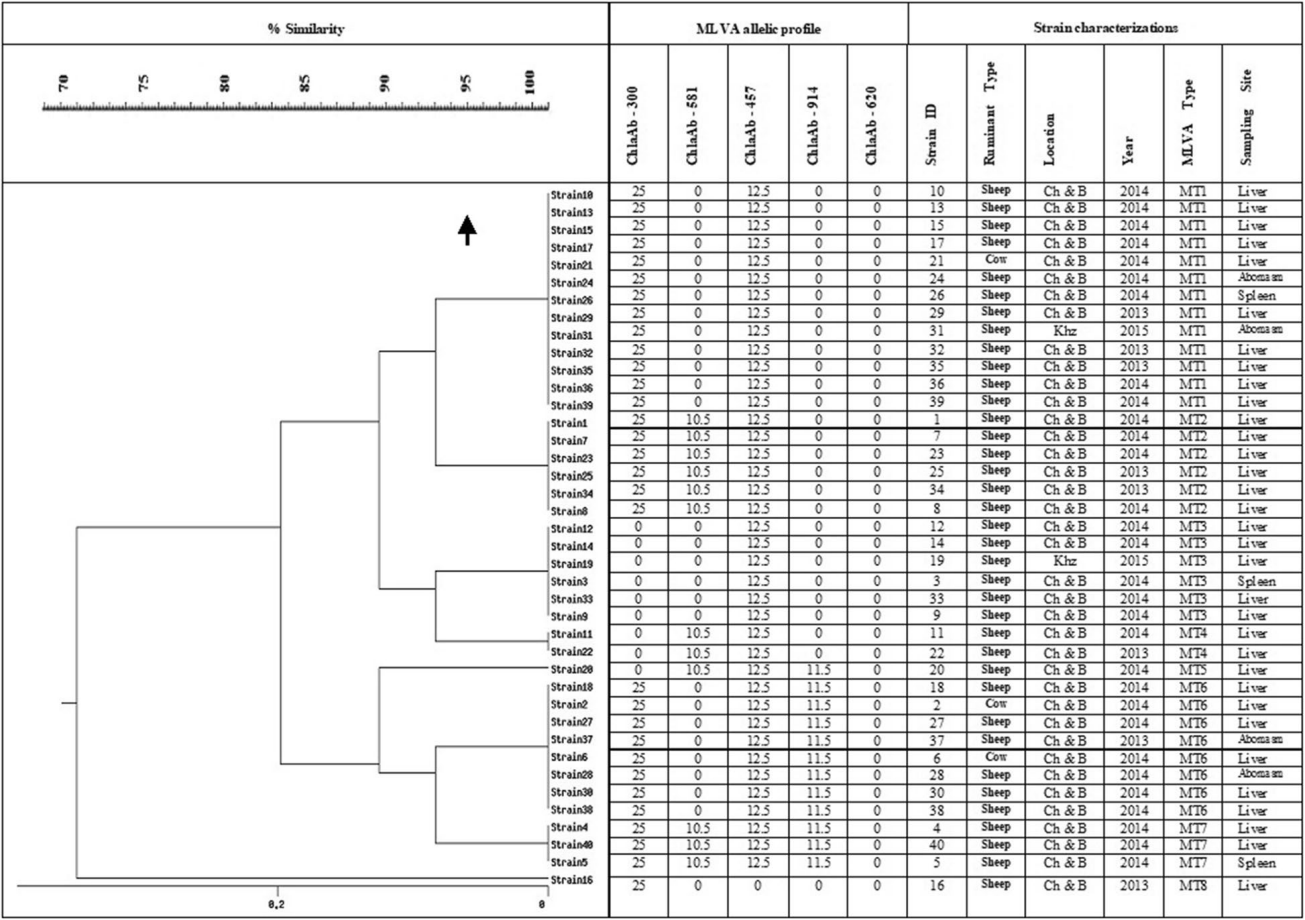
Dendrogram of *C. abortus* based on MLVA analysis. Results are compared using the Dice method and clustered by UPGMA.

The results of MLVA analysis in 40 strains (one strain from each aborted fetus) are shown in Figs. 6 and 7. Generally, 32% of *C. abortus* strains (13/40) were grouped in MT1 as the main MT type. Three bovine strains from Charmahal/Bakhtiari were grouped in MT1(one strain) and MT6 (two strains). The bovine strains were similar to the clusters from sheep in terms of size (base pair). Following MT1 as the main type, MT6 type with 8 strains and MT2 and MT3 types with 6 strains were in the second and third places, respectively. The minimum number of strains was in MT4 and MT5 groups with merely 2 and 1, respectively (Table 5). All MLVA types were detected in the collected strains from Charmahal/Bakhtiari but only 2 types (MT1 and MT3) were reported in those from Khuzestan. The ChlaAb_457 genome (CAB398 coding for a histone-like protein) with 385 bp fragment size was detected in 39 (97.5%) of the *C. abortus* strains while the ChlaAb_620 genome (CAB541 coding for a conserved membrane protein) with 163 bp fragment size was not detected in any of the strains. According to the attained results, the most and the least variations were detected in MT7 strains and MT3 strains, respectively. In the present study, the basis of the classification of the strains in different clusters was genetic similarity, accounting for more than 95%. The genetic affinity observed among the strains in cluster 1 was reported to be higher than those in other clusters despite the differences in sampling time and geographical areas. Three bovine strains were settled in 2 different clusters. Also, 2 sheep strains in Charmahal/Bakhtiari were detected as singleton with different patterns of VNTRs and sampling time. Significantly, strains 21 and 31 with different sampling time and geographical area were grouped in MT1. In Fig. 6, additional data about variations in the loci subjected to MLVA in the studied strains are demonstrated.

**Table 5:** Genotypic classification according to animal species

Animal species	MT1	MT2	MT3	MT4	MT5	MT6	MT7	MT8
Sheep	12 (92.3%)	6 (100%)	6 (100%)	2 (100%)	1 (100%)	6 (75%)	3 (100%)	1 (100%)
Cow	1 (7.69%)	–	–	–	–	2 (25%)	–	–
total	13 (32.5%)	6 (15%)	6 (15%)	2 (5%)	1 (2.5%)	8 (20%)	3 (7.5%)	1 (2.5%)

## Discussion

Abortion is economically important in many types of ruminants in Europe, North America, Africa, and Asia. *C. abortus* gives rise to reproductive disorders, inflammation of the epididymis, pneumonia, arthritis and conjunctivitis. It is even found in the feces of healthy sheep and goats and could also be a zoonotic risk for pregnant women [21, 22, 28].

In several studies, molecular techniques, such as various PCR assays and real-time PCR based on different goal genes (*omp*A, IGS-S*rRNA*, 16S*rRNA*, *pomp*90–3, *pomp*90–4, and 16 s–23 s spacer region) have been used for the detection or phylogenic analysis of *C. abortus* strains in the aborted fetuses of ewes [23, 29–31]. Certainly, these methods have different sensitivity and specificity.

The role of Chlamydiosis in different types of animals in Iran, particularly in the 2 studied provinces, has been investigated by serologic and molecular tests; however, due to the complications of the culture method and common clinical signs, isolation has never been the focal point of research. Undoubtedly, isolation and genotyping of *C. abortus* were conducted by the present research, is the first attempt to carry out in-depth studies in this context in Iran.

In the present study, for the primary detection of *C. abortus* in the infected samples, real-time PCR was used. By this method, the probability of an error in the differentiation of *C. psittaci* and *C. abortus* or missing the coinfection with 2 species is the least. Based on the results, the Chlamydial infection of ruminants in the present research was 34.1% whereas the *C. abortus* infection revealed to be 100%, which are considerably high percentages. It is worth noting that in spite of the equal number of collected samples in 2 studied provinces, the detected positive samples in Charmahal/Bakhtiari outnumbered those in Khuzestan. This could be related to more traditional training of ruminants in Charmahal/Bakhtiari compared to Khuzestan.

It is necessary to show the relationship between pathogenic and non-pathogenic strains as well as the distribution patterns of the pathogenic strains in the region due to being regarded as determining factors to control pathogenic strains. Pathogenic typing can assist us in understanding the pathogenic relationship between the prevalent types and their sources (human or animal) and the differentiation of current and new infections [32].

The efficacy of the MLVA method has been confirmed by several researchers for the purpose of typing different microorganisms. Laroucau et al. (2009) used 8 selected loci in 9 standard strains of *C. psittaci* in birds for typing 150 clinical *C. psittaci* isolates with different hosts in various areas. They stated that this method has a higher diagnostic value and more sensitivity compared with serotyping by *omp*A gene sequencing. Genotyping of *C. abortus* strains based on five selected loci (ChlaAb_457, ChlaAb_581, ChlaAb_620, ChlaAb_914, and ChlaAb_300) has been shown to be appropriate for clinical samples [6].

Li et al. (2015), studied 135 samples (9 aborted fetuses, 126 vaginal swabs) of yak papulation in China. *C. abortus* infection was reported in 9 aborted fetuses (100%) and 30 vaginal swabs (23.81%). MLVA typing of 4 isolated strains in this study was grouped in MT2 [33].

Also, Malal and Turkyilmas in Turkey studied the material samples of abortion in 267 cattle, 380 sheep, 70 goats and 13 water buffaloes. *C. abortus* infection was reported in 11.9% of the studied samples and as a result of MLVA typing, MT2 was found as the dominant genotype (93.1%). Certainly, genotype 3, 4 and 5 were involved in the infections [34].

In the present research, a study on genetic linkage among *C. abortus* strains in 2 provinces of Iran was carried out for the first time, using MLVA. According to our findings, there was roughly 80 to 100% similarity between sheep and bovine *C. abortus* strains in the 2 studied regions, respectively. The MLVA typing method, based on the analysis of 5 VNTR loci, enabled the clustering of 40 *C. abortus* strains into 8 genotypes; this showed genotyping diversity among *C. abortus* strains which included 8 different MLVA types (MT1–8). Variation of MTs in Charmahal/Bakhtiari (MT1- MT8) was more than that of Khuzestan (MT1, MT3) province, with due attention to study of different number of positive samples in two areas. There was a close genetic linkage among the strains from Khuzestan. Furthermore, the occurrence of common clones from Khuzestan and Chaharmahal/Bakhtiari indicated a genetic similarity in *C. abortus* strains from these regions and between the sheep and cattle strains. This provides evidence for the transmission risk of *C. abortus* ruminant infection from these areas. In the same vein, Seth-Smith et al. (2017) reported that the diversity within *C. abortus* as compared to other species is much lower; moreover, the genome of *C. abortus* is highly stable [13]. Therefore, suitable strategies should be employed to curb the possible spread of infection due to *C. abortus* in sheep and cattle in the studied regions. Due to the proximity of these 2 provinces and the migration of animals between them, these findings were expected relatively.

In the current research, MT1 was reported as the dominant type but in another research regarding *C. abortus* in Asia (Turkey and China), MT2 turned out to be dominant. Considering the common border between Iran and Turkey and also the probability of animal interchange, augmented similarity in MLVA type is expectable. Any investigation regarding *C. abortus* genotyping in areas where Turkey shares border with West Azarbayejan province can shed light on such variations. The exact reason behind different predominant *C. abortus* MLVA type in areas with no common borderline such as China or any European country could be due to various management and hygienic factors.

Siarkou et al. (2015) reported 7 types in the MLVA genotyping of 94 ruminant *C. abortus* isolates, with the highest repetition variation occurring in MT2 [35]. The correlation between repetitive locus number and the virulence of strains was mentioned in the Laouracu et al. (2008) study [20]. In the present research, 3 strains were demonstrated with 4 repetitions of 5 loci which are more acute compared with other strains. A survey of the repetitive profile (5 studied loci) in 40 isolates showed that the ChlaAb_457 and ChlaAb_620 loci appeared in 97.5 and 0% of the isolates, respectively.

The collection of samples was carried out over a period of 3 years (2014–2016); therefore, the time difference can also be investigated in the present research. The highest and the lowest variations were observed in MT1 and MT7, respectively. The isolates in the other types (MT2, MT3, MT4, MT5, and MT6) were a combination across 3 years which could be indicative of the genetic relationship between these types.

## Conclusions

For the first time, the prevalence of *C. abortus* in ruminants of Iran was detected by real- time PCR. Based on results, *C. abortus* could be reported as important agents responsible for ruminant abortion in Khuzestan and Charmahal/Bakhtiari provinces. In addition to, economic loos aspect of Chlamydial abortion, geographical situation of Khuzestan province and its important role in animal transport between internal provinces and neighbor countries such as Iraq, are strong reasons for special attention to review of prevention management in this area. Comparison with neighbor countries such as Turkey, *C. abortus* abortion percentage in small ruminants was 2–3 times more in Iran, which could be related to more restrict managements in this country. By genotyping of *C. abortus* in this research for the first time in Iran, high genotype diversity from MT1 to MT8 was detected. Although, diverse with another studies in different part of the world (e.g. Turkey, France, UK), MT1 was reported as dominant genotype in studied areas. In spite of, significant aspects of this research, necessity of study of *C. abortus* abortion rate in humans and typing of *C. abortus* in another areas of Iran is stable and recommendable.

## Data Availability

The datasets used and/or analyzed during the current study are available from the corresponding author on reasonable request.

## Abbreviations

BSA: Bovine serum albumin
*C.abortus*: *Chlamydia abortus*
*C. psittaci*: *Chlamydia psittaci*
dpi: day post-infection
MLVA: multiple loci variable number of tandem repeats analysis
MT: MLVA type
PBS,: Phosphate Buffer Saline
PBST: PBS + 0.2% Tween20
Pmp: polymorphic membrane protein
RPMI 1640 medium: Roswell Park Memorial Institute Medium
SPG: Sucrose Phosphate Glutamate
TMH: transmembrane head protein
UPGMA: Unweighted Pair-Group Method with Arithmetic
VNTR: variable number of tandem repeats
